# Tunable angle-independent refractive index sensor based on Fano resonance in integrated metal and graphene nanoribbons

**DOI:** 10.1038/srep29984

**Published:** 2016-07-21

**Authors:** Meiyan Pan, Zhaoxing Liang, Yu Wang, Yihang Chen

**Affiliations:** 1 Guangdong Provincial Key Laboratory of Quantum Engineering and Quantum Materials, School of Physics and Telecommunication Engineering, South China Normal University, Guangzhou 510006, China; 2 Department of Applied Physics, The Hong Kong Polytechnic University, Hong Kong SAR, China

## Abstract

We propose a novel mechanism to construct a tunable and ultracompact refractive index sensor by using the Fano resonance in metal-graphene hybrid nanostructure. Plasmon modes in graphene nanoribbons and waveguide resonance modes in the slits of metal strip array coexist in this system. Strong interference between the two different modes occurs when they are spectrally overlapped, resulting in a Fano-type asymmetrically spectral lineshape which can be used for detecting the variations of ambient refractive index. The proposed sensor has a relatively high figure of merit (FOM) over 20 and its sensing performance shows a good tolerance to roughness. In addition to the wide range measurement enabled by the electrical tuning of graphene plasmon modes, such ultracompact system also provides an angle-independent operation and therefore, it can efficiently work for the detection of gas, liquid, or solids. Such optical nanostructure may also be applied to diverse fields such as temperature/pressure metering, medical detection, and mechanical precision measurement.

Exhibiting an asymmetric profile of spectral lineshape, Fano resonance (FR) was first experimentally observed by Beutler^1^ and theoretically interpreted by Ugo Fano^2^. It was described as a result of the quantum interference between a discrete excited state of an atom and a continuum sharing the same energy level. Due to the analogous superposition principle of waves, a number of phenomena in classical optics, such as Wood’s anomaly^3^, can also be explained by FR. The FR in optics arises from the overlap of a narrow discrete resonance with a broad spectral line. With the rise of plasmonics, FR has been observed in numerous nanoscale plasmonic structures over the past few years. A manner to obtain FR is to break the geometric symmetry of nanostructures^4^, by which sharp subradiant modes (high-order oscillations) were excited and then interacted with the broad superradiant mode (dipole). Another compelling approach is by using grating structures^5^ where localized particle plasmons and optical waveguide modes coexist. Hybrid nanostructures such as Fanoshells and mismatched nanorods^5^ can also meet the FR requirements. In particular, plasmonic planar oligomers^5^, where FRs were excited via the hybridization of dipole modes of each constituent nanoparticle, displayed superior sensitivity to environmental changes.

Fano-type transmission/reflection based on localized surface plasmon resonance (LSPR)^4^,^5^,^6^,^7^ was widely investigated among SPR sensing applications where an ultrahigh sensitivity over 10000 nm/RIU has been experimentally achieved^7^. However, one major deficiency that limits the applications of these FR-based structures is the lack of dynamically active tunability. Graphene, a two-dimensional carbon material with outstanding electrical and optical properties can, however, be used to fill this gap. In addition to its abundant applications such as terahertz laser^8^, absorber^9^, modulator^10^, graphene is also an excellent candidate for sensing applications^11^,^12^ due to its large specific surface area and high carrier mobility. Compared with the surface plasmons in metal, graphene surface plasmons (GSPs) show both higher confinement and less loss and they can be effectively tuned by electrical doping. Modulation of FR in plasmonic dolmen structures was experimentally achieved by the absorption of graphene sheet^12^. However, such an absorption-based modulation can only change the transmittance but not the lineshape or the frequency of FR. Recently, tunable Fano-type lineshape was obtained through the interaction of two coupled SPs supported by metal and graphene gratings respectively^13^. Nevertheless it is well known that the coupled SP modes can only be excited with phase-matching techniques. The difference between the wave vectors of light and coupled SPs was mainly compensated by the additional wave vector provided by the periodic structure and the projection along the interface of the momentum of photons^14^. Therefore, plasmonic devices based on the coupled SP modes require a spatial period with the same order of magnitude of the resonant wavelength, resulting in inevitably large dimensions at infrared and terahertz regime, and their performance strongly depends on the incident angle. In contrast to the coupled SP modes, the waveguide-resonance (WR) mode localized in slits of metal stripes can offer a broader angle-independent transmission band. Furthermore, the width and the period of the slits are much smaller than the resonant wavelength, which enable operation in sub-wavelength structures^15^.

In this paper, we propose an ultracompact FR-based metal-graphene hybrid nanostructure for detecting the refractive index variations of surrounding dielectrics. The sensor operates at mid-infrared wavelengths and its performance is electrically tunable and angle-insensitive. A schematic representation of the metal-graphene hybrid nanostructure is depicted in Fig. 1. Graphene and silver nanoribbons are deposited, with a sub-wavelength period, on a PMMA/Ag/PMMA substrate to generate spectrally overlapped GSP and waveguide-mode resonance. The interaction of these two SPs can lead to a Fano-type asymmetric lineshape used for sensing the changes in refractive index. Our proposed mechanism for the design of refractive index sensors can also be applied in far infrared and terahertz regimes.

## Results and Discussion

**Figure 2:**
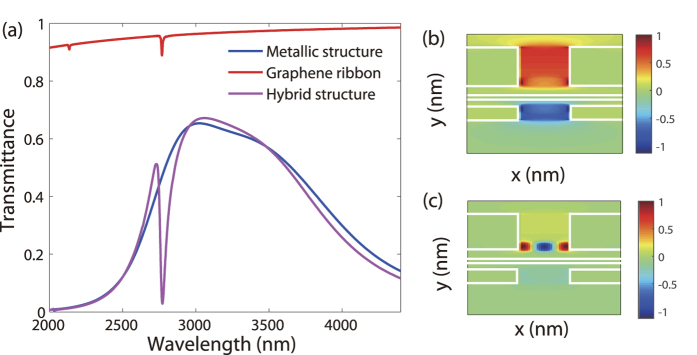
(**a**) Transmittance for 42 nm-wide graphene ribbons on an effective substrate (red), metallic structure without graphene (blue), and entire hybrid structure (mauve). Normalized electric field distribution of (**b**) the metallic structure without graphene at *λ* = 3001 nm and (**c**) the entire hybrid structure at *λ* = 2770 nm. Fermi energy of graphene is 0.74 eV.

We first consider the metallic nanostructure without introducing the graphene ribbons. As mentioned before, TM-polarized normal incidence can excite two different resonant modes in metal strip array, the coupled SP mode at strip surface and the WR mode in the slits of strips, at rather different frequencies^15^. To make the WR mode dominate in the discussed frequency range (Fig. 2(b)), the width (*W*_*M*_), period (*p*), heights of the upper (*h*_1_) and and bottom (*h*_2_) silver strips are set as 358 nm, 410 nm, 180 nm, and 40 nm, respectively. To achieve optimal transmission performance, other structural parameters are *d*_1_ = 55 nm, *d*_2_ = 35 nm, *t* = 20 nm^16^. TM-polarized light flows through the subwavelength slits due to the excitation of WR which provides an absorption band of the incident energy. The absorbed energy then passes through the middle Ag film by coupling, and finally transfers to emitting energy by means of the metal slits at the bottom. Finite-difference time-domain (FDTD) method was used to calculate the transmission spectra, where parameters of silver are obtained from experimental data (Palik)^17^. As shown by the blue line in Fig. 2(a), a broad transmission band appears.

It has been demonstrated^18^ that GSPs (discrete resonances) can be excited under TM-polarized illumination, resulting in sharp dips in the transmission spectrum (red line in Fig. 2(a)). In our simulations, graphene was treated as an extremely thin layer with an effective dielectric constant *ε*_*g*_ = 2.5 + *i*σ_g_/*ε*_0_*ω*Δ, where *ε*_0_ is the permittivity in vacuum, *ω* is the circular frequency of incident light, and Δ is the thickness of graphene (Δ = 0.5 nm). Under the conditions of low temperature and high doping (i.e. 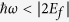, where *E*_*f*_ represents the Fermi energy of graphene), the graphene conductivity is dominated by intraband transition and thereby can be simplified as 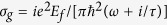
19, where *e* is the electron charge, 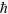 is the reduced Plank constant, and *τ* is the relaxation time. In our considered wavelength range, the relatively low photon energies (compared with *E*_*f*_) means that the graphene conductivity obtained from the simplified semiclassical model should agree well with that calculated from random phase approximation (RPA) which can give the accurate expression of the graphene dielectric constant^19^.

When graphene ribbons are placed inside the slits of the metal strip array, GSPs may be excited by the WR and then interact with the WR. In particular, the spectral overlap of these two plasmon resonances may result in a Fano-type lineshape. To realize such a spectral overlap, the width (*W*_*G*_) of the graphene ribbons is set as 42 nm, which is achievable via current fabrication technology^20^. As shown in Fig. 2(a) (mauve curve), a Fano-type lineshape appears in the transmission spectrum for the Ag-graphene hybrid nanostructure. Note that the plasmons interaction also leads to slightly shifts of the transmission peak and dip corresponding to the two different SPs, respectively. Consequently, the response of the graphene ribbons is equivalent to that on an effective substrate whose refractive index is 1.785. More interestingly, due to the energy localization by the WR, the resonant energy can be fully transferred to GSPs and then be extremely confined by graphene, as shown in Fig. 2(c). Hence, the transmission dip originated from the FR effect is much deeper than that produced by only GSP, suggesting a better sensing performance can be realized using the hybrid structure.

**Figure 3:**
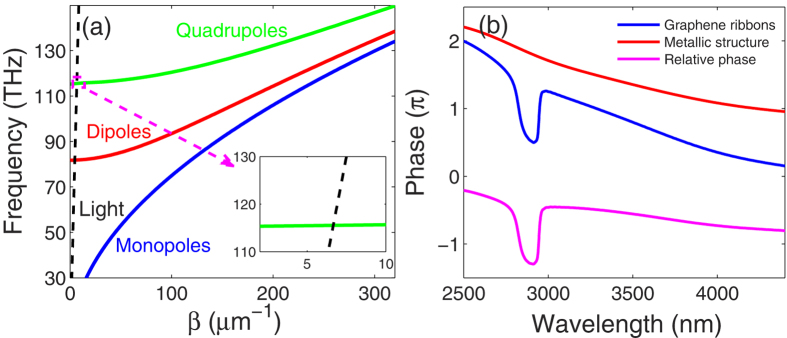
(**a**) Dispersion curves of graphene ribbons (*E*_*f*_ = 0.74 eV, *W*_*G*_ = 42 nm) on an effective homogeneous substrate. Inset: zoomed-in image of the intersection point of light line and the quadrupole mode dispersion curve of GSPs. (**b**) Transmission phase of the metallic structure without graphene (red), the graphene ribbons (blue) and the relative phase of the GSPs to the WR (marve).

In the graphene ribbons, resonant oscillation modes are supported by free carriers with restricted motions^21^. The electric field on graphene exhibits a standing wave profile^22^ with a wave number *k*_*x*_ ≈ *mπ*/*W*_*G*_, where *m* represents the number of the nodes. By applying Maxwell’s equations and proper boundary relations, the dispersion relations of SPPs in the graphene ribbons with dielectric substrate can be obtained as


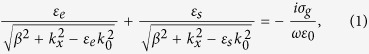


where *β* represents the wave vector of the propagating SPPs, *ε*_*e*_/*ε*_*s*_ is the relative permittivity of envioronment/substrate, and *k*_*0*_ is the wave vector of light in vacuum. As shown in Fig. 3(a), all plasmon resonances, except for the monopole mode, can be excited by normal illumination at the corresponding intersection points of the dispersion curves and the light line. Here, we consider the third-order resonance, i.e., quadrupole mode. Note that the interactions among the plasmonic oscillations in the neighboring ribbons are neglected because of large separations^23^.

As a key factor of plasmon interference, the transmission phases of the metallic structure and the graphene ribbons were examined. For an oscillator that produces a Lorentzian lineshape transmission profile, its phase varies approximately by *π* when the frequency travels across the resonance^24^. As shown in Fig. 3(b), due to the broad transmission band, the *π*-change of the transmission phase of WR in the metal slits takes place slowly. By contrast, the sharp and discrete plasmon resonances of the graphene ribbons exhibit a phase jump of *π* at the resonance frequencies. Consequently, the relative phase of GSP to WR shows analogous *π*-phase jumps. As a result, a minimum and a maximum of the transmittance emerge due to the destructive and constructive interference at the two sides of the corresponding resonance frequency, respectively.

**Figure 4:**
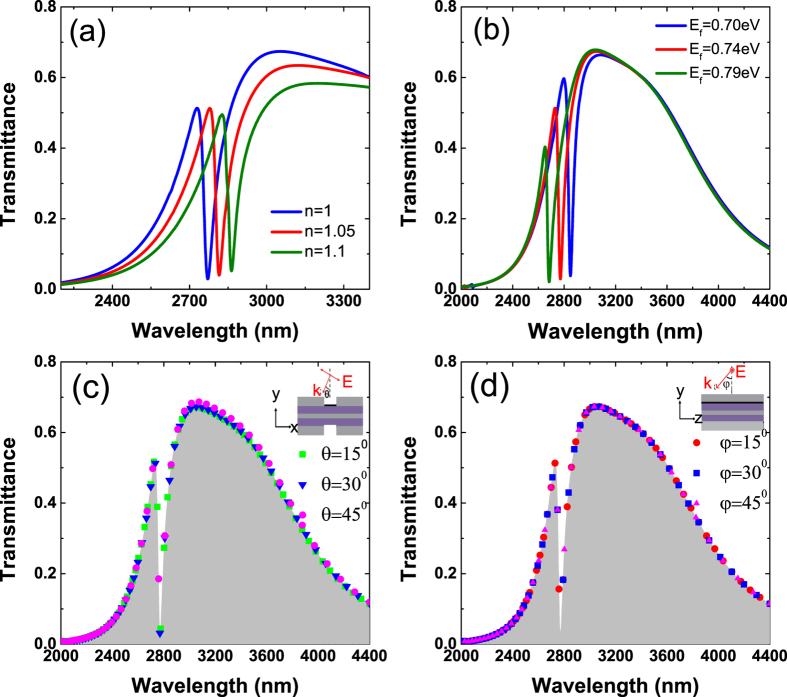
Simulated normal-incidence transmission spectra for the Ag-graphene hybrid structure. (**a**) With different ambient refractive indices (*n*) when the Fermi energy of graphene *E*_*f*_ = 0.74 eV and for the structure with different *E*_*f*_ when *n* = 1. (**c**,**d**) Show the transmission spectra for the hybrid nanostructure at different incident angles. The gray area indicates the performance under normal incidence. Insets show the polarization and incident angle configurations.

The frequency of the FR depends on the sharp GSP oscillation. As the ambient refractive index *n* increases, the propagation wavevectors corresponding to the same frequency increases, i.e., the dispersion curves shown in Fig. 3(a) descend. Then the intersections of the light line and the dispersion curves decline, indicating the decreasing resonant frequencies. Hence, the Fano transmission dip exhibits an obvious redshift as the ambient refractive index increases (Fig. 4(a)). This feature provides an excellent scheme of nanoscale sensing for the refractive index variations of environmental dielectrics such as gas. Among the discussions on the refractive index sensors, figure of merit (FOM) is a key assessment criteria and it is defined as^25^


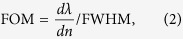


where *dn* represents a small index change which leads to a spectral shift of a resonance *dλ*, FWHM is the full width at half maximum. Such a definition of FOM was usually applied to the general Lorentz-type symmetric lineshape. To evaluate the performance of the FR-based sensor, *dλ* is defined as the average shift of the Fano peak and dip and FWHM is equal to the wavelength difference between the peak and dip^4^. Based on the simulations, the FOM value of our proposed configuration is determined to be about 26 which does not reach the highest level among existing plasmonic sensing systems^26^,^27^. However, the performance of the metal-based plasmonic systems is difficult to be tuned without changing the geometric dimensions.

For the considered graphene-based hybrid system, the relatively high sensitivity sensing may fail for the ambient dielectric with too small/large refractive index as the transmission dip shifts to a position out of the operation regime. Fortunately, this can be overcome by the electrical tuning of GSP. The Fermi energy of graphene can be dynamically tuned by applying bias voltage *V*_*g*_, with an approximate relationship 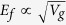
28. Figure 4(b) shows that the FR transmission peak and dip both blue shift with the increasing Fermi energy, which is consistent with the discussion above. The influence of the Fermi energy on the GSP resonance suggests that the measurement range of the proposed sensor can be efficiently broadened by means of electrical tuning.

In addition, our nanoscale sensor also shows excellent insensitivity to the angle of incidence. P-polarized plane wave is used and the transmission spectra of the Ag-graphene hybrid nanostructure at different incident angles were calculated (shown in Fig. 4(c,d)). The angle-independent property of the transmission dip is mainly inherited from the characteristics of GSPs. As mentioned above, the oscillations of GSPs depend on the environmental refractive index, the width of the graphene ribbons, and the graphene conductivity (determined by the Fermi energy). For the WR of the metallic strip array, the transmitted intensities only depend on the width of the slits^15^. As a result, the transmission profile of the hybrid nanostructure remains invariant with the change of the incident angle.

**Figure 5:**
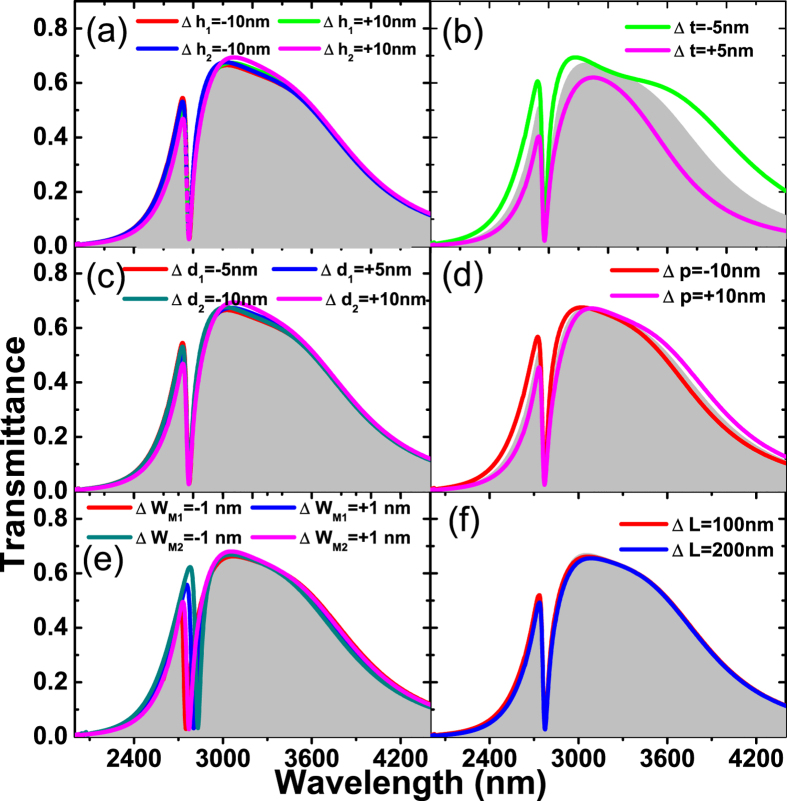
Transmittance for the hyrid structure by varying the geometric parameters. (**a**) Heights of the upper and bottom silver nanostrips, (**b**) thicknesses of the upper and bottom PMMA films, (**c**) thickness of the Ag film, (**d**) period of the Ag-graphene hybrid grating, (**e**) widths of the slits, (**f**) lateral displacement between the two Ag gratings, respectively. Positive/negative value means that the corresponding parameter is larger/smaller than that of the optimized design, whose features are illustrated by the gray area.

As far as fabrication is concerned, accurate dimensions of the hybrid nanostructure are challenging to meet, geometrical errors may degrade the sensing performance. In fact, this problem can be automatically overcome by our configuration. As shown in Fig. 5, the variations of the heights of silver nanostrip arrays (Fig. 5(a)), the thickness of PMMA layer (Fig. 5(b)), the thickness of the middle silver film (Fig. 5(c)), or the period of the hybrid grating (Fig. 5(d)) has little effect on the sensing performance because the position of the FR transmission dip and the FWHM value remain almost unchanged. The Fano transmission dip shifts as the widths of the slits vary, which does not bring obvious change of the FOM value, as shown in Fig. 5(e). More significantly, the proposed sensor has an excellent tolerance on the misalignment of the periodic structures on the top and bottom. As shown in Fig. 5(f), the Fano feature in the hybrid structure with large lateral displacement between the top and bottom gratings remains invariant. Such a stable and robust FR transmission profile of our proposed sensor is efficient for practical applications.

## Conclusions

In summary, we report a novel design of a tunable, angle-independent refractive index sensor with high sensitivity based on the Fano resonance originated from the interference between the surface plasmon in the graphene nanoribbons and the waveguide-mode resonance in the slits of metal stripes. Although the configuration is complicated and the sensing performance is a little worse than the highest level among plasmonic sensing systems, the variations of the incident angle and the fabrication errors have little influence on the sensing performance. We also showed that the sensing performance can be electrically tuned by adjusting the bias voltage on the graphene nanoribbons without any geometrical changes, making adjustable wide-range sensing possible. Since the environment refractive index is affected by temperature as well as pressure, the proposed structure could also be used as a thermometer or a barometer. Furthermore, the sensitive response of the hybrid structure to the local change in refractive index, resulting from the specific capture of molecules/biomolecules at the sensor surface, could enable the sensing of concentration of molecules/biomolecules.

## Methods

### Derivation of the dispersion relation for GSP in graphene ribbons

To reveal the physical mechanism for the excitation of graphene surface plasmons (GSPs), we study the dispersion relation of the GSPs. Assume that the graphene ribbons have a finite width of *W*_*G*_ along *x* direction. Figure 2(c) shows that *E*_*x*_ has a standing wave-like profile within the graphene ribbons. Therefore, in addition to the propagating wave vector (*β*) of GSPs, another component of wave vector is produced in the graphene nanoribbons by the oscillation modes of free carriers with restricted motions: *k*_*x*_ ≈ *mπ*/*W*_*G*_, where *m* represents the number of nodes.

The thickness of the graphene ribbons is too small and are neglected in the following derivation for the dispersion relationship of GSP in a graphene ribbon. According to the Maxwell’s Equations


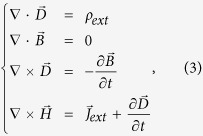


the macroscopic quantities 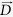 (the dielectric displacement), 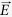 (the electric field), 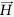 (the magnetic field), and 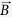 (the magnetic induction) are linked with the external charge *ρ*_*ext*_ and the current densities 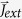, the time harmonic electromagnetic field yields


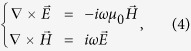


where *ω* is the circular frequency of incident light, *μ*_0_ and *ε* represent permeability and permittivity of the dielectrics. As a characteristic of SPP, the electromagnetic field has a form of 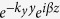, where *β* represents the propagating constant on graphene and *k*_*y*_ indicates the exponential amplitude decay along *y* direction. *E_x_* component is neccessary for the exctiation of GSPP, and we assume a TM-polarized incidence to the considered system along y direction. Equation (4) hence can be expanded to


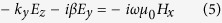



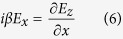



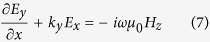



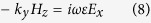



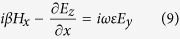



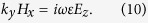


It should be noted that *E*_*x*_, *E*_*y*_, *E*_*z*_, *H*_*x*_ and *H*_*z*_ are included in Eqs (5, 6, 7, 8, 9, 10) even though the incident wave contains only *E*_*x*_, *E*_*y*_ and *H*_*x*_. This is the result that the charge distribution on graphene, which is regarded as graphene surface charge, changes the characteristic of the electromagnetic field near the graphene layer. For example, the non-homogeneous electron distribution on graphene along z-axis produces the *E*_*z*_ component of the electromagnetic field. However, *H*_*y*_ remains zero because it is independent of the surface charge. Then combining Eqs (6), (9) and (10), we obtain a wave equation


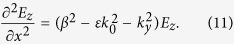


Since 
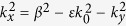
, a general solution of *E*_*z*_ can be written as


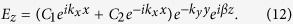


Combining Eq. (12) with Eq. (3), all components of the electromagnetic field can be solved. For instance, substituting Eq. (9) into Eq. (5), we have 
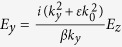
. The components of the electromagnetic field are


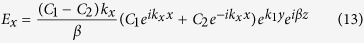



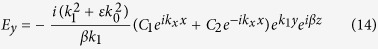



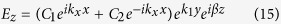



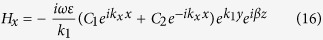



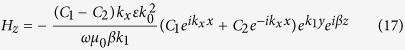


for the half space with *y *< 0 and


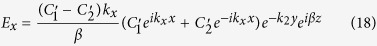



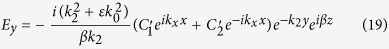



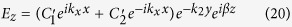



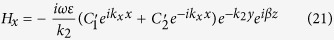



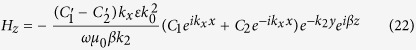


for the half space with *y* > 0. *k*_*s*_ ≡ *k*_*y,s*_(*s* = 1, 2) is the wave vector component perpendicular to the interface of the two dielectric media. According to boundary relations, when crossing an interface, the tangential component of electric field is continuous while the transverse component of magnetic field is affected by the surface current as the following equation:


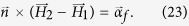


Note that


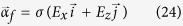


and the surface conductivity σ is provided by the graphene sheet (σ_*g*_). By applying the boundary relations to Eqs (8) and (9), we obtain


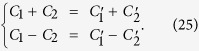


Combining Eqs (20), (22), (24) and (25), we get the final result, the dispersion relation of SPPs propagating in graphene ribbons at the interface of two dielectric spaces:


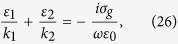


where 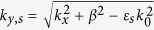 (*s* = 1, 2) and *k*_*x*_ = *mπ*/*W*_*G*_.

### Fabrication process of the proposed refractive index sensor

**Figure 6:**
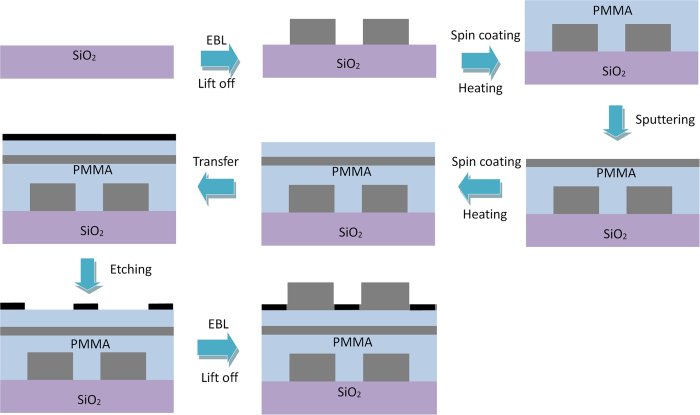
Schematic of fabrication process of the proposed refractive index sensor.

Such a complicated structure is experimentally achievable. Figure 6 illustrates the detailed fabrication proposal: the lower silver grating is fabricated by electron beam lithography (EBL) and lift-off technique first^29^. Next, a 55 nm-thick PMMA is evenly covered on the grating by spin coating method and then solidified by heating (8 nm-thick PMMA layer has been experimentally realized^30^). Then, a silver thin film is deposited with magnetron sputtering technique^31^ before another PMMA film is coated on. After these, a graphene sheet is transferred onto the surface of the PMMA thin film^32^ and then is etched into nanoribbon array by electron-beam induced nano-etching^33^. Finally, the upper silver grating is generated by EBL and lift-off technique where a resist (for example, ZEP^34^), which is much more sensitive than PMMA, and a small dose of electron beam should be used.

## Additional Information

**How to cite this article**: Pan, M. *et al*. Tunable angle-independent refractive index sensor based on Fano resonance in integrated metal and graphene nanoribbons. *Sci. Rep.* **6**, 29984; doi: 10.1038/srep29984 (2016).

## Figures and Tables

**Figure 1:**
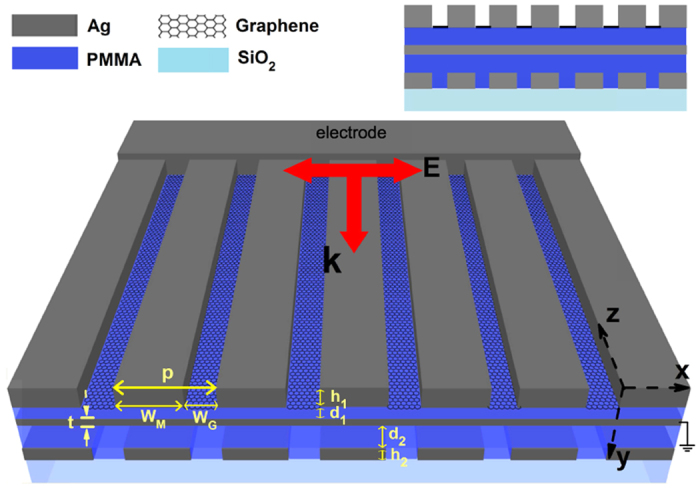
Schematic of the Ag-graphene hybrid structure where graphene and silver nanoribbons are alternatively deposited on the top surface of a PMMA/Ag/PMMA substrate. The structural parameters are: *h*_1_ = 180 nm, *d*_1_ = 55 nm, *h*_2_ = 40 nm, *d*_2_ = 35 nm, *p* = 410 nm, *W*_*M*_ = 358 nm, *W*_*G*_ = 42 nm and *t* = 20 nm. Inset is the cross section of the design.
